# Exclusive breastfeeding and associated factors among mothers in Gozamin district, northwest Ethiopia: a community based cross-sectional study

**DOI:** 10.1186/s13006-017-0121-1

**Published:** 2017-07-10

**Authors:** Melkamu Tamir Hunegnaw, Lemma Derseh Gezie, Alemayehu Shimeka Teferra

**Affiliations:** 1 Department of Human Nutrition, College of Medicine and Health Science, University of Gondar, P.O Box, 196 Gondar, Ethiopia; 2 Department of Epidemiology and Biostatistics, Institute of Public Health, College of Medicine and Health Sciences, University of Gondar, P.O Box, 196 Gondar, Ethiopia

**Keywords:** Exclusive breastfeeding, Gozamin district, Ethiopia

## Abstract

**Background:**

Exclusive breastfeeding is defined as feeding infants only breast milk, be it directly from breast or expressed, except drops or syrups consisting of vitamins, mineral supplements or medicine. Exclusive breastfeeding is one of the essential actions for infant development and survival. However, the prevalence of exclusive breastfeeding in Ethiopia has been estimated at 52% which is far less than the World Health Organization (WHO) recommendations. Moreover, there are inconsistencies among estimates in different districts of the country. Therefore, this study aimed to assess the prevalence and associated factors of exclusive breastfeeding among mothers in Gozamin district, northwest Ethiopia.

**Methods:**

Using the simple random sampling technique, seven kebeles (lowest administrative units) were selected as the primary sampling unit of the district. Sample mother-infant pairs were then selected using the systematic random sampling technique that involved our moving from house to house in each village. Data were collected from 506 mother-infant pairs using interviews. Factors associated with exclusive breastfeeding were determined using logistic regression. The measure of association used was the odds ratio, and statistical tests with *p*-values of less than 0.05 were considered as statistically significant.

**Results:**

In this study, the prevalence of exclusive breastfeeding among mothers was 74.1% (95% CI 70.80, 79.10). For government employee mothers, the odds of exclusive breastfeeding were reduced by half compared to housewives (AOR 0.49, 95% CI 0.26, 0.94). Mothers who did not receive breastfeeding counseling after delivery were 0.43 times less likely to practice exclusive breastfeeding compared with mothers who received the services (AOR 0.43, 95% CI 0.25, 0.72). Mothers who gave birth at health institutions were more likely to practice exclusive breastfeeding.

**Conclusion:**

Even though the estimated prevalence is relatively high, more effort to meet WHO recommendations is still necessary. Therefore, we suggest health institutions encourage hospital birthing and increase breastfeeding counseling after delivery, and employers needs to give longer maternity leave to improve exclusive breastfeeding practice.

## Background

In order to achieve optimal growth, development and health, the World Health Organization (WHO) and the National Nutrition Programme (NNP) of Ethiopia recommend that infants should be exclusively breastfed for the first six months of life, which means that infants should receive only breast milk except prescribed medications, vitamins, and minerals [1, 2]. The Government of Ethiopia strives to address under-nutrition through the Lifecycle Approach which includes ensuring that newborns are breastfed within one hour of birth and that exclusive breastfeeding (EBF) continue for the first six months, followed by adequate complementary feeding [2]. Breastfeeding has a significant role in improving nutrition, education, and maternal and child health [3].

Though studies showed that EBF has a potential to reduce under- five mortality by 11.6%, the prevalence of EBF is still relatively low globally, as in sub Saharan Africa with a magnitude of 35% and 22–33%, respectively [4]. According to WHO, 90% EBF practice strongly reduces the incidence of infant mortality due to pneumonia [5]. The prevalence of EBF varied from country to country. For example, in Canada 10.4% of mothers exclusively breastfed [6], while in the Republic of Congo 2.8% did so [7]. On the contrary the prevalence of EBF was higher in developing countries than in developed ones, 49% in Timor, Asia, and 35.4% in Korea [8, 9].

Studies conducted on EBF in Ethiopia reported a variety of findings ranging from 29.3% in Addis Ababa [10] to 81.1% in Dubti town, northeast Ethiopia [11]. The prevalence also varied from locality to locality within the region, and was 50.1% for Motta district [12], and 79% for Azezo district [13] both of which are to the northwest of the Amhara Regional State, respectively. In addition to the disparities of EBF practice in different places of Ethiopia, it seems that according to reports from different authors, the country might not reach the target set to be achieved by 2019/2020 which is 93% [14]. Understanding the factors that hinder this target point might be helpful to carry out an intervention in improving the practice.

Different studies indicated various factors that could influence EBF practices. A study conducted in Nova Scotia, Canada, showed that EBF was associated with maternal education and family income [6], but these were not the predictors for EBF practice in a study in Indonesia [15]. Maternal age was a significant factor in the study done in sub-Saharan Africa, but not so in Indonesia [15, 16]. A study carried out in Italy indicated that antenatal care was a significant factor for EBF practice [17], but not the study done in Nigeria [18].

In Ethiopia, maternal age was not an independent predictor of EBF according to a study done in Dabat, northwest Ethiopia [19], but it was a significant factor in Debre Berhan, central Ethiopia [20]. In the study in Dabat, family income was not a significant predictor for EBF practice [19], while it was statistically associated with EBF according to a study done in Awi Zone, northwest Ethiopia [21].

As can be seen from the literature in Ethiopia, there were inconsistencies of prevalence of EBF as well as factors associated with it. This is what motivated us to conduct this study in an area where no similar study has been conducted to date.

## Methods

### Study design and period

A community based cross-sectional study was conducted to examine the prevalence and associated factors of exclusive breastfeeding practice among mothers who had infants aged between 6 and 12 months, from 1 March to 20 March 20 2016.

### Study setting and participants

The study was conducted in Gozamin district, located 300 km northwest of Addis Ababa, the capital of Ethiopia. The district has 30 kebeles (lowest local administrative units). The district has a total population of 153, 151 of whom 30, 982 are women in the reproductive age group (15–49 years). There also 20,743 under-five age children and 4769 infants less than one year of age. The district has six health centers and 26 health posts that provide health services to the community [22].

### Sample size, sampling technique, and procedures

The single population proportion formula was used to determine sample size, assuming a 95% of confidence level, 5% margin of error, and 29% prevalence of exclusive breastfeeding [10]. As we have used two stage sampling, a design effect of 1.5 is considered in determining the sample size. Finally, a contingency of 10% was used to account for non-response during data collection. Therefore, the final sample size was estimated to be 506 mother-infant pairs.

Among the 30 kebeles in the district, seven were selected using the simple random sampling technique to achieve the primary sampling units*.* The total sample size was allocated to the seven kebeles proportionally, based on the number of mother-infant pairs in each kebeles received from the district health office. Sample mother-infant pairs were selected using the systematic random sampling technique with a sampling interval of three. In order to source infants eligible for the study, we moved from village to village of each selected kebeles, assessing each and every household. Out of the first three households with infants eligible for the study, the second household was selected randomly as a random start. Then, out of households with infants aged between 6 and 12 months, every three mother-infant pair were included in the sample.

### Data collection and quality control

Data were collected using interviewer administered structured questionnaire which was designed to assess EBF practice and associated factors. Each mother with an infant aged between 6 and 12 months was interviewed in order to get data regarding EBF practice and factors associated with it. The questionnaire included socioeconomic, obstetric, health, and health service related characteristics.

In order to maintain the quality of data, the questionnaire was translated from English to Amharic and back to English for consistency. Training that included field practice was given to data collectors and supervisors in kebeles which were not included in the main study. On-site supervision was carried out by the investigators and supervisors, and feedback was given.

### Variables and operational definitions

The dependent variable of the current study is exclusive breastfeeding, and an infant fed only breast milk except taking vitamins, mineral supplements, or medicines until six months [1] was included as exclusive breastfeeding. Infants who had received exclusive breastfeeding correctly were coded ‘1’ in the SPSS during analysis, while those that had not been EBF correctly were coded ‘0’.

The independent variables considered in this study include maternal age, marital status, maternal education, maternal occupation, monthly family income, antenatal care, breastfeeding counseling, place and mode of delivery, and HIV status of mothers.

Maternal age was categorized into four groups (≤ 24, 25–29, 30–34, and ≥35 years), and the younger age was taken as a reference category in the logistic regression analysis. Marital status single was coded ‘0’ and married/union was coded as ‘1’. Maternal education ‘1’ was the code for mothers who were non-educated or who attended informal education and the rest were coded as ‘0’. Housewife mothers were coded as ‘1’ and government and private workers were encoded as ‘0’. Family monthly income was categorized into five levels, and the lowest level was taken as a reference category. Place of delivery was categorized into three (at home, government hospital, and health center), mothers who delivered at home were coded as ‘1’, the rest were encoded as ‘0’. Mothers who attended antenatal care, received breastfeeding counseling services, and had breastfeeding experience were coded as ‘1’ and their respective counterparts were coded as ‘0’. Mothers with Human Immunodeficiency Virus (HIV) positive mothers were coded as ‘0’ and HIV negative mothers as ‘1’. A health post is the lowest health facility structure in Ethiopian health tier system.

### Statistical analysis

Data were entered, coded and cleaned, using Epi-info version 7.0 statistical software and then transferred to SPSS software version 20 for further data processing and analysis. Text descriptions, tables, charts, and graphs were used to describe the relevant findings of the study.

The Crude Odds Ratios (COR) with a 95% confidence interval (CI) were estimated in the binary logistic regression analysis to assess the association between each independent variable and the outcome variable, and to select candidate variables for the multivariate logistic regression analysis. Because there were relatively a large number of independent variables considered in this study, we had to screen them using the bivariate logistic regression to minimize the chance of multicollinearity in the multivariate logistic regression. Thus, only those independent variables with a *p* - values of 0.20 or less in the bivariate logistic regression were included in the multivariate logistic regression to get the adjusted effect of each covariate [23].

Adjusted Odds Ratio with a 95% confidence interval were estimated to assess the strength of the association. Variables with *p* - value less than 0.05 in the multivariate logistic regression analysis were considered as significant and independent predictors of exclusive breastfeeding practice.

## Results

### Sociodemographic characteristics

Out of a total of 506 eligible mother-infant pairs, 478 participated in this study with a response rate of 94.4%.The mean age of mothers was 28.62 ± 4.95 SD years. Four hundred twenty-five mothers (88.1%) were married, 461 (96.4%) were Orthodox Christians, and 472 (98.7%) were Amhara in ethnicity. More than half (51.9%) of the mothers were housewives and 141 (29.5%) completed secondary school (Table 1).

**Table 1:** Sociodemographic characteristics of mothers in Gozamin district, northwest Ethiopia (*n* = 478)

Characteristics	Number	Percent
Ethnicity of mother		
Amhara		
Tigray	472	98.7
SNNP	4	0.8
Religion of mother	2	0.5
Orthodox	461	96.4
Muslim	7	1.5
Protestant	10	2.0
Sex of infant		
Male	259	54.2
Female	219	45.8
Maternal age (in years)		
≤ 24	88	18.4
25–29	198	41.4
30–34	128	26.8
≥ 35	64	13.4
Marital status		
Married	425	88.1
Divorced	13	2.7
Widowed	44	9.2
Number of children		
≤ 3	414	86.6
> 3	64	13.4
Maternal education		
Informal and not educated	91	19.0
Primary	89	18.6
Secondary	141	29.5
Certificate, and above	157	32.8
Educational status of husband		
Informal and none educated	88	18.4
Primary	70	14.6
Secondary	115	24.1
Certificate and above	205	42.9
Maternal occupation		
House wife	248	51.9
Government employee	106	22.2
Private employee	113	23.6
Private organization employee	11	2.3
Family monthly income (ETB)		
≤ 650	76	15.9
651–1400	87	18.2
1401–2350	103	21.5
2351–3550	81	16.9
≥ 3551	131	27.4

*ETB* Ethiopian Birr

### Maternal and infant health service utilizations

Of all the study participants, 450 (94.1%) of the mothers attended antenatal care during pregnancy. Out those who had antenatal care, 333 (69.7%) mothers received breastfeeding counseling. Four hundred thirty eight (91.6%) mothers had their infants with vaginal mode of delivery **(**Table 2).

**Table 2:** Health and health service characteristics of mothers in Gozamin district, northwest Ethiopia (*n* = 478)

Characteristics	Number	Percent
Antenatal care		
Yes	450	94.1
No	28	5.9
Breastfeeding counseling during pregnancy		
Yes	333	69.7
No	145	30.3
Place of birth/delivery		
At home	53	11.1
Government hospital	321	67.1
Government health center, post	104	21.8
Mode of delivery		
Vaginal	438	91.6
Cesarean section	40	8.4
Attendant of the delivery		
Health profession	430	90.0
Relative/friends	48	10.0
Breastfeeding counseling after delivery		
Yes	365	76.4
No	113	23.6
Breastfeeding experience		
Yes	273	57.1
No	205	42.9
Maternal HIV status		
Positive	27	5.6
Negative	451	94.4

### Exclusive breastfeeding by other characteristics

Among all mothers who participated in the study, nearly three-fourths (74.1%) breastfed their infants exclusively during the first six months of age (95% CI 70.8, 79.1). In this study, among mothers who had male infants, 73% practiced exclusive breastfeeding, while 77.2% of the mothers who had female infants did so. Similarly, among mothers who were attended by health professionals during delivery, 78.4% practiced EBF while only 43.8% of the mothers attended by relatives or friends breastfed their infants exclusively (Table 3).

**Table 3:** Exclusive breastfeeding by different factors for mothers in Gozamin district, northwest Ethiopia (*n* = 478)

Characteristics	Exclusive breastfeeding	
	Number	Percent
Exclusive breastfeeding		
Yes	355	74.1
No	123	25.9
Sex of infant		
Male	189	73
Female	169	77.2
Delivery attendant		
Health professional	377	78.4
Relative/friend	21	43.8
Birth interval		
First pregnancy	157	81.8
27–38 months	31	75.6
≥ 39 months	170	69.4
Number of children		
1–3	317	76.6
≥ 4	41	64.1

### Factors associated with exclusive breastfeeding

In the bivariate logistic regression, sex, marital status, educational status, HIV status of the mothers, and counseling about breastfeeding during antenatal care were not found to have statistically significant association with EBF at *p* - value of 0.2. However, variables including maternal age, family monthly income, occupation of the mother, place of delivery, counseling about breastfeeding after delivery, and five other variables were included in the multivariable logistic regression model.

When each independent variable was adjusted for other variables, occupation of mother, place of delivery, and counseling about breastfeeding after delivery were found to be statistically significantly associated with exclusive breastfeeding at a 95% confidence level and a *p* - value of 0.05.

Government employed mothers reduced the odds of EBF practice almost by 50% (AOR 0.49, 95% CI 0.26, 0.94) compared to housewives. Similarly, mothers who did not receive counseling about breastfeeding after delivery reduced the odds of EBF by 57.4% compared to their non-counseled counterparts (AOR 0.43, 95% CI 0.25, 0.72). The odds of EBF for mothers who delivered at the government hospital was two-fold (AOR 2.01, 95% CI 1.02, 3.94) compared to mothers who delivered at home (Table 4).

**Table 4:** Bivariate and multivariate logistic regression analysis of exclusive breastfeeding among mothers in Gozamin district, northwest Ethiopia (*n* = 478)

Variables	Exclusive breastfeeding		Crude Odds Ratio (95% CI)	Adjusted Odds Ratio (95% CI)
	Yes (%) No (%)			
Maternal age (in years)				
≤ 24	72 (81.8)	16 (18.2)	1	1
25–29	155 (78.3)	43 (21.7)	0.80 (0.42, 1.52)	0.87 (0.44, 1.73)
30–34	88 (68.8)	40 (31.2)	0.49 (0.25, 0.94)	0.52 (0.26, 1.06)
**≥** 35	43 (67.2)	21 (32.8)	0.46 (0.21, 0.97)	0.57 (0.25,1.27)
Marital status				
Married/in union	317 (75.3)	104 (24.7)	1	1
Single	41 (71.9)	16 (28.1)	0.84 (0.45, 1.56)	0.81 (0.38,1.74)
Maternal education				
No education & Informal	62 (68.1)	29 (31.9)	1	1
Primary education	70 (78.7)	19 (21.3)	1.72 (0.88, 3.37)	1.41 (0.65, 3.05)
Secondary education	110 (78.0)	31 (22.0)	1.66 (0.92, 3.01)	0.93 (0.45, 1.91)
Certificate and above	116 (73.9)	41 (26.1)	1.29 (0.73, 2.27)	0.76 (0.33, 1.77)
Husband’ education				
Informal education	59 (67.0)	29 (33.0)	1	1
Primary education	56 (80.0)	14 (20.0)	1.97 (0.94, 4.10)	1.55 (0.64, 3.77)
Secondary education	91 (79.1)	24 (20.9)	1.86 (0.9, 3.01)	1.23 (0.50, 3.02)
Certificate and above	152 (74.1)	53 (25.9)	1.32 (0.75, 2.33)	1.02 (0.41, 2.56)
Maternal occupation				
House wife	180 (72.6)	68 (27.4)	1	1
Government employee	75 (70.8)	31 (29.2)	0.91 (0.55, 1.51)	0.49 (0.26, 0.94)*
Private work	103 (83.1)	21 (16.9)	1.85 (1.07, 3.20)	1.69 (0.95, 2.99)
Monthly family income (in Birr)				
≤ 650	53 (69.7)	23 (30.3)	1	1
651–1400	60 (69.0)	27 (31.0)	0.96 (0.49,1.90)	0.91 (0.44,1.86)
1401–2350	83 (70.6)	20 (19.4)	1.80 (0.90, 3.59)	1.93 (0.92,4.03)
2351–3550	60 (74.1)	21 (25.9)	1.24 (0.62, 2.49)	1.22 (0.57,2.58)
≥ 3551	102 (77.9)	29 (22.1)	1.53 (0.80, 2.89)	2.15 (0.98, 4.72)
Antenatal care				
Yes	341 (74.8)	109 (24.2)	1	1
No	17 (60.7)	11 (39.3)	0.49 (0.23, 1.09)	0.65 (0.27, 1.56)
BF counseling during ANC				
Yes	253 (76.0)	80 (24.0)	1	1
No	105 (72.4)	40 (27.6)	0.83 (0.53, 1.29)	0.90 (0.52, 1.55)
Place of delivery				
Home	26 (49.1)	27 (50.9)	1	1
Government hospital	243 (75.7)	78 (24.3)	3.23 (1.78, 5.87)	2.01 (1.02, 3.94)*
Health center, post	89 (85.6)	15 (14.4)	6.16 (2.86, 13–28)	4.56 (2.01, 10.37)*
Mode of delivery				
Cesarean section	34 (85.0)	6 (15.0)	1.99 (0.82,4.87)	2.02 (0.79, 5.16)
Vaginal	324 (74.0)	114 (26.0)	1	1
BF counseling after delivery				
Yes	290 (79.5)	75 (20.5)	1	
No	68 (60.2)	45 (39.8)	0.39 (0.25, 0.60)	0.43 (0.25, 0.72)*
Maternal HIV status				
Positive	21 (77.8)	6 (22.2)	1.18 (0.47, 3.01)	1.08 (0.37, 3.14)
Negative	337 (74.7)	114 (25.3)	1	1
Breastfeeding experience				
Yes	194 (71.1)	79 (28.9)	1	1
No	164 (80.0)	41 (20.0)	1.63 (1.06, 2.51)	1.26 (0.76, 2.09)

*Significant at *p -* value of ≤0.05, BF- breastfeeding, ANC- antenatal care

## Discussion

This study has attempted to assess the magnitude of exclusive breastfeeding practice and associated factors during the first six months of infant life among mother-infant pairs in Gozamin district, northwest Ethiopia. The prevalence of EBF was 74.1%. This finding was greater than that of a study conducted in Dabat district, northwest Ethiopia, whose prevalence of EBF was 30.7% [24]. It was also higher than the national level prevalence of 52% reported by Ethiopian Demographic and Health Survey (EDHS) in 2011 [22]. This difference could be the result of efforts and multi-sectoral collaborations that have been made by the Government of Ethiopia on child nutrition since 2013 [14], while the studies mentioned above used data collected before the implementation of these revised national nutrition programs. In addition, the national study included samples from the less developed and pastoral regions with less health service coverage, which might have resulted in less awareness about exclusive breastfeeding.

Regarding factors that could affect EBF, occupation of the mother, place of delivery, and breastfeeding counseling after delivery were statistically significantly associated with EBF practice. Accordingly, government employed mothers were less likely to practice EBF compared to housewife mothers. This finding is in line with those of studies done in the rural communities of northwest Ethiopia [24], eastern region of Ghana [25], Kinshasa [7], and Saudi Arabia [26]. Perhaps this is because employed mothers have no time to exclusively breastfeed their infants, or they have short maternity leave to stay with and establish breastfeeding their newborn babies, or they lack convenient locations to breastfeed at their working places. On the contrary, a study done in Bangladesh showed that mothers working outside their houses were more likely to practice exclusive breastfeeding. The possible explanation for this difference might be that in Bangladesh most women take their babies to their work places [27].

In this study, mothers who delivered at health institutions were more likely to practice exclusive breastfeeding compared to mothers who delivered at home. These findings were similar to a study done at Dabat Health and Demographic Surveillance System site in northwest Ethiopia [24] and Ghana [28]. This might be so because mothers who gave birth at health institutions could have more chance to receive obstetric and postnatal care, nutritional education and counseling on the benefits of breastfeeding, correct positioning, and attachment.

Exclusive breastfeeding in this study is significantly associated with breastfeeding counseling after delivery. This result is supported studies done in Debre Berhan district, central Ethiopia [20], Addis Ababa public health centers, Ethiopia [10], and Kilimanjaro Region, northern Tanzania [29]. One possible explanation for the observed association could be that breastfeeding counseling after delivery might have helped mothers to improve their maternal knowledge regarding exclusive breastfeeding.

In our study, antenatal care visits were not significantly associated with EBF practice. However, in studies conducted in Dabat, northwest Ethiopia [24], among urban women at Addis Ababa public health centers, Ethiopia [10], and Timor-Leste [8] indicated that mothers who had antenatal care visits had a better practice of exclusive breastfeeding. The possible reason for this difference might be that in this study mothers who attended antenatal care did not receive breastfeeding counseling.

### Limitations of the study

In our study, the age of the infants in the mother-infant pairs was between 6 and 2 months. This period could be assumed to be long enough to result in a recall bias of either over or underestimating the finding of the work.

## Conclusion

The incidence of exclusive breastfeeding in this work was relatively high compared to other previous studies. A mothers’ occupation, giving birth at health facilities, and the provision of counseling about breastfeeding after delivery were significantly associated with EBF practice. Therefore, we suggest that health institutions encourage hospital birthing and increase breastfeeding counseling after delivery. Employers need to consider giving longer maternity leave to improve exclusive breastfeeding practice.

## Abbreviations

AOR: Adjusted Odd Ratio
BF: Breastfeeding
COR: Crude Odd Ratio
EBF: Exclusive Breastfeeding
EDHS: Ethiopian Demographic and Health Survey
ETB: Ethiopian Birr
NGO: Non Governmental Organizations
UNICEF: United Nations Children’s Fund
WHO: World Health Organization
